# Relationship Between Congestive Heart Failure and the Dietary Index of Gut Microbiota: Information From the National Health and Nutrition Examination Survey 2007–2018

**DOI:** 10.1002/fsn3.71386

**Published:** 2025-12-31

**Authors:** Yu Fu, Hanfei Wang, Yulu Jiang, Jiana Yang, Jianquan Yu, Anru Cao, Yue Liu, Jie Wu

**Affiliations:** ^1^ School of Public Health Shenyang Medical College Shenyang Liaoning China; ^2^ Liaoning Medical Functional Food Professional Technology Innovation Center Shenyang Medical College Shenyang Liaoning China; ^3^ Mr. Selenium (Jilin) Biotechnology co., Ltd Shenyang Liaoning China; ^4^ Shangshan Innovation Research (Shenyang) Technology co., LTD Shenyang Liaoning China; ^5^ School of Traditional Chinese Medicine Shenyang Medical College Shenyang Liaoning China

**Keywords:** congestive heart failure, diet, dietary index of gut microbiota, NHANES

## Abstract

The Gut Microbiota Dietary Index (DI‐GM) assesses connections between diet and microbial diversity. The morbidity and mortality of congestive heart failure are currently increasing, but the existing evidence regarding the relationship is scarce between congestive heart failure and the gut microbiota dietary index. This research looked at the possible connection between dietary markers of the microbiota in the gut and CHF. The research incorporated information from individuals 20 years of age or older, regardless of whether they had congestive heart failure. It provided that their gut microbiota dietary index data was complete, as sourced from the National Health and Nutrition Examination Survey (NHNAES) carried out from 2007 until 2018. The affiliation between the gut microbiota dietary index and congestive heart failure was evaluated with multivariate logistic regression (MLR), limited cubic spline analysis, subgroup analysis, interaction analysis, and sensitivity analyses. This study examined 8671 participants, among whom 277 were diagnosed with congestive heart failure (CHF), while the remaining 8394 showed no signs of the condition. Once logistic regression has been used to account for all relevant variables, the results of the research showed that CHF risk and DI‐GM scores were inversely related. Specifically, those that scored best on the DI‐GM had a considerably reduced chance of developing CHF, with OR: 0.0002 and 95% CI: 0.000–0.001, in contrast to those with lower scores. A comprehensive analysis employing restricted cubic spline (RCS) methods discovered a nonlinear relationship between the incidence of CHF and DI‐GM. Subjects with elevated DI‐GM scores showed a lower likelihood of developing congestive heart failure than those with poorer scores, with body mass index acting as a mediator in this connection. These findings could open fresh avenues for CHF treatment strategies, though additional longitudinal studies will be necessary to confirm whether DI‐GM directly influences CHF risk.

## Graphical abstract

In this study, which explored the relationship between DI‐GM and CHF, we found that higher DI‐GM scores were associated with a reduced risk of CHF compared with participants with lower DI‐GM scores, and the association remained significant even after adjusting for covariates, which could provide evidence for prospective studies and provide literature support for CHF prevention.

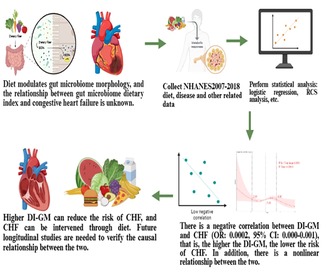

**Abbreviations**
95% CI: 95% confidence intervals
AHA/ACCF: the American Heart Association/American College of Cardiology guidelines
BMI: body mass index
CDAI: Composite Dietary Antioxidant Index
CHD: coronary heart disease
CHF: congestive heart failure
CVD: cardiovascular diseases
DI‐GM: dietary index for gut microbiota
HDL‐C: high‐density lipoprotein cholesterol
IL‐1β: interleukin‐1 beta
LDL: low‐density lipoprotein
NHANES: National Health and Nutrition Examination Survey
OR: ratio of ratios
PIR: poverty income ratio
RCS: restricted cubic spline
SCFAs/SCFA: short‐chain fatty acids
Th17: T Helper 17
TMAO: trimethylamine N‐oxide

## 1 Introduction

The microbiota in the gut is complicated and large ecosystem which includes many microorganisms. For example, in the case of bacteria, fungi, and viruses. The gut microbiome significantly influences human health, impacting immunity, metabolism, intestinal barrier function, and neurobehavioral traits (Kase et al. 2024). There is a link between the development of some diseases (e.g., obesity, gastrointestinal disorders, metabolic syndrome) and imbalance or dysregulation of the gut flora (Hevia et al. 2014), while many clinical studies have found that gut microbes and cardiovascular illness is linked to the formation of TMAO (Organ et al. 2016).

One of the many variables affecting gut microbiota is nutrition, which has a significant impact on human health. The Western eating pattern is distinguished by its high fat and carbohydrate intake and low fiber content—promotes significant gut microflora imbalance (Tomasello et al. 2016). Short‐term diet modifications (intake of particular nutrients, foods, or specialized diets) can swiftly and markedly alter gut microbiota (Partula et al. 2019). High‐fat diets can increase bifidobacteria in the gut flora, resulting in the activation of Th17 cells that promote inflammation (Beam et al. 2021), however, the gut flora associated with a ketogenic diet can reduce the concentrations of pro‐inflammatory Th17 cells within the gastrointestinal tract, which reduces inflammation within the gut microbiota (Ang et al. 2020). The Mediterranean diet emphasizes plant‐based foods packed with fiber and healthy fats while minimizing animal products and saturated fats. This nutritional approach appears to support gut microbiome balance while optimizing the body's metabolic processes (García‐Montero et al. 2021). Research indicates that the classic Mediterranean diet could help prevent heart failure (Tuttolomondo et al. 2019). A better prognosis for people with chronic heart failure has been linked to diets rich in fiber, prebiotics, and polyphenols because they alter the gut's microbiota's makeup and boost the microbial metabolite synthesis (Li et al. 2025). The breakdown of intestinal dietary fiber microorganisms produces short‐chain fatty acids (SCFAs), in order to maintain cardiovascular well‐being. Both clinical studies involving human participants and research using animal models have consistently linked SCFA levels to key risk factors for cardiovascular disease (Chen, Peng, et al. 2023).

The Dietary Index of Gut Microbiota (DI‐GM) serves as a measurement for evaluating the influence of dietary habits on the makeup and role of the gut microbiota (Huang et al. 2025). Kase et al. (2024) analyzed 106 studies to pinpoint 14 key nutrients and foods in the scope of DI‐GM. Nutrients or food items recognized for their positive effects are believed to enhance both the gut microbiota species' alpha and beta diversity indexes. The total levels of SCFAs, which include butyrate, acetate, propionate, as well as isobutyrate, have increased in tandem with this improvement. A balanced ratio of the phyla Thicket to Anthrobacterium is also noted. In this context, the advantageous components encompass whole grains, nutritional fiber, cranberries, avocados, broccoli, coffee, green tea, chickpeas, soybeans, and fermented dairy products; on the other hand, contrasting effects are noted with various other substances. Meats in red consumption, handled meat consumption, sophisticated grain consumption, and diets heavy in fat (defined as making up 40% or more of energy) adversely affects gut microbiota, representing harmful components of the diet (An et al. 2024), effectively illustrating the connection between food quality and the variety of gut flora.

The final development of all cardiac illnesses is congestive heart failure (CHF), a complicated clinical condition, a diverse syndrome, and a worldwide public health issue (Xu et al. 2021). An estimated 64.3 million people around the world have CHF (Groenewegen et al. 2020). The total prevalence of heart disease (which includes stroke, coronary heart disease, and congestive heart failure) inside the America's United States is recorded at 9.3%, with a noted increase in incidence as age advances for both males and females (Kadier et al. 2022). Not only are conventional risk factors including high blood pressure, excessive body weight, elevated blood sugar levels, and tobacco now well recognized (Chen, Lin, et al. 2023). However, there is mounting evidence that the development of different CVDs is influenced by the gut flora (Li et al. 2025). Therefore, this study's primary objective was to look at the connection between dietary markers linked to gut microbiota and congestive heart failure, employing data sourced from NHANES. This research aims to provide fresh perspectives on the management of CHF and strengthen the comprehension of the exchanges between microbiome in the gut and this health condition.

## 2 Method

### 2.1 Data Sources

The twenty‐four‐hour NHANES dietary recall data was employed to produce DI‐GM. A nationwide survey is NHANES that is cross‐sectional conducted biennially, making use of a multistage, using a stratified sampling technique to choose participants, with the primary aim of assessing the United States' population's nutritional status and overall health (Kase et al. 2024). Before the initiation of interviews, informed consent, documented in writing, was secured from every participant involved in the study and The National Centre for Health Statistics ensured that every piece of data was anonymized before being made publicly available. The survey included a comprehensive household interview, a physical examination, and laboratory evaluations carried out in a mobile screening facility specifically designed for this initiative (Han et al. 2023). The participants involved in this research were adults who were at least 20 years old, selected to look into the ties between the gut microbiota dietary index and CHF. This research was conducted by the ethical standards described in the Helsinki Declaration. Our investigation used anonymised data that was available to the public. The procedures concerning data collection were looked at and authorized by the Research Ethics Review Board of the National Centre for Health Statistics (Han et al. 2023).

### 2.2 Study Population

**FIGURE 1:**
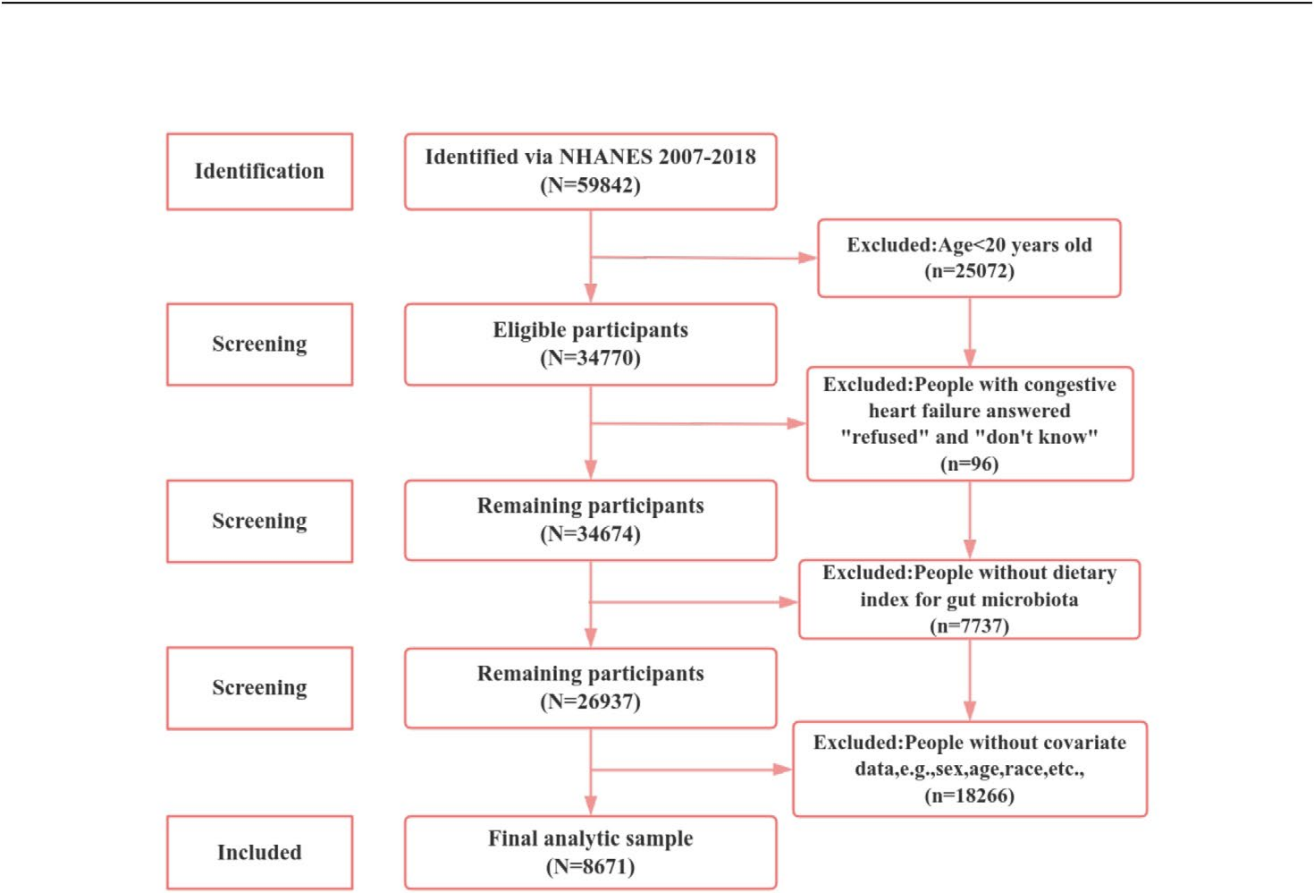
Participant selection flow diagram.

In this enquiry, We excluded anyone under the age of 20 and included everyone above the age of 20 (*n* = 25,072). Additionally, participants diagnosed with congestive heart failure who selected “refused” or “don't know” were excluded (*n* = 96), alongside individuals with incomplete data regarding the DI‐GM (*n* = 7737). Furthermore, we excluded participants lacking essential covariate information, including demographic data, BMI, smoking habits, use of alcohol, and medical history (*n* = 18,266). We use the direct deletion approach, which has the benefits of ease of use and quick computation time, for all missing values. In the end, 8671 individuals were kept for examination. Figure 1 provides specifics on the grounds for inclusion and removal.

### 2.3 Study Variables and Covariates

Congestive heart failure was assumed to be present in those who replied “yes” to the query, “Have you ever received a diagnosis of heart failure?,” in the MCQ160B NHANES database. Sex, age, race, education, marriage, PIR (≤ 1, 1–3, > 3), BMI (≤ 124.9, 25–29.9, > 30 kg/m^2^), hyperlipidemia, hypertension, diabetes, coronary heart disease, angina pectoris, heart disease, stroke, cancer, sleep (≤ 7, 7–8, > 8 h/night), smoking (do you currently smoke?) and alcohol consumption (yes or no). Our criteria for identifying hyperlipidemia were LDL cholesterol (< 40 mg/dL), triglycerides (≥ 200 mg/dL), total cholesterol content (≥ 240 mg/dL), and HDL‐C (males < 40 mg/dL and females < 50 mg/dL).

### 2.4 Predictor Variables

We calculated the DI‐GM utilizing 24‐h meal remember information from NHANES. The gender‐specific median intake served as the basis for the DI‐GM score. Every part was given a score of either 0 or 1, and the individual scores were summed to produce a comprehensive score for the DI‐GM. Each participant was given a score of 1 if their consumption of all the advantages dietary component exceeded the median for their sex and their consumption of unfavorable components fell below the median for each sex. Conversely, a score of zero was tasked with those whose advantageous component intake was below the median and whose unfavorable component intake was above it. These separate element scores were then totaled to calculate a DI‐GM rating, which could between 0 and 14. A better gut microbial profile is indicated by a higher DI‐GM rating. The specific DI‐GM ratings originated from Professor Zheng's NHANES data platform (https://www.zstats.cn/software/nhanes_data/nhanes_data_2.0.2.4.0/).

### 2.5 Methods of Statistical Analysis

R version 4.4.3 and SPSS 27 were applied to every statistical analysis. Following the guidelines specified in the NHANES analysis guide, we included weights of sampling and considered principal units of sampling along with division of labour. Weighted means with corresponding standard deviations were used to represent continuous variables in the analysis of baseline characteristics, whilst weighted percentages were used to represent categorical data. To evaluate the differences in characteristics among various participant groups, continuative figures were analyzed using one‐sample *t*‐tests and chi‐squared tests to assess categorical data.

People who take part were separated according to their DI‐GM values into four quartiles: Q1 (ranges from 0 to 4), Q2 (ranges from 5 to 6), Q3 (ranges from 6 to 11), and Q4 (ranges from 11 to 12). Weighting was applied before conducting logistic regression analysis, RCS analysis, mediation analysis, and sensitivity analysis. The connection between CHF and DI‐GM was investigated using logistic regression with several variables. The risk of CHF was assessed using probability ratios (ORs) and 95% confidence intervals (95% CIs). Only DI‐GM and congestive heart failure were examined in the crude model, which failed to take any factors into consideration. Model 1 only controlled for three covariates: sex, age, and race, whereas Model 2 did so for all of them, including sex, age, race, education, and marriage. A discrepancy that is statistically significant is shown by a *p* value of < 0.05 on both sides.

To look into the possible nonlinear connections between DI‐GM and heart failure, we carried out an analysis employing restricted cubic splines (RCS) while thoroughly controlling for pertinent covariates. Results that are important in terms of statistics (*p* < 0.05) were obtained for the non‐linearity test, confirming a nonuniform relationship. This analysis was implemented in R using the following packages: “ggplot2,” “ggpubr,” “survival,” “splines,” “foreign,” and “rms.” Investigations were conducted on the connection between CHF and DI‐GM, using three segmental inflection point‐based logistic regression models.

Furthermore, to investigate how this association varied across different demographic groups, we conducted subgroup analyses and tested for interactions. For visual representation, we generated forest plots using R's “forestplot” and “ggplot2” packages. Finally, we not only included albumin as a covariate in the original data to evaluate the association between DI‐GM and CHF, but also resurveyed the data from 2007 to 2018. With other covariates remaining unchanged, New covariates, such as physical activity, total energy intake, chronic kidney disease, and related drugs (e.g., diuretics, ACEi/ARB, and beta‐blockers), were added. A total of 7197 participants were included to explore the association between DI‐GM and CHF to verify the consistency of the results.

## 3 Results

### 3.1 Basic Details

In the NHANES continuous examination conducted between 2007 and 2018, we examined the history of congestive heart failure in a total of 8671 participants. Of these 8671 participants with complete data, 277 were found to have congestive heart failure and 8394 were found not to have congestive heart failure. The prevalence of CHF was 3.46% (*n* = 143) and 2.95% (*n* = 134) in women and men, respectively. The subjects' average age without CHF was 49.57 ± 17.50 years and 67.04 ± 12.40 years for those with CHF. Those with CHF tended to be older, less educated, had unhealthy lifestyle habits (smoking, alcohol consumption, and sleep deprivation), and had a higher predominance of factors of risk, such as heart disease, angina pectoris, coronary artery disease, diabetes mellitus, and hypertension compared to non‐CHF participants. The basic qualities of the research participants appear in Table 1.

**TABLE 1:** Basic characteristics of the participants.

Variable	Total (*n* = 8671)	Yes (*n* = 277)	No (*n* = 8394)	*p*
Age	50.12 ± 17.63	67.04 ± 12.40	49.57 ± 17.50	< 0.001
High‐density lipoprotein cholesterol (mg/dL)	54.29 ± 15.97	49.75 ± 16.76	54.44 ± 15.92	< 0.001
Low‐density lipoprotein cholesterol (mg/dL)	114.21 ± 35.61	101.56 ± 38.60	114.63 ± 35.43	< 0.001
Total cholesterol (mg/dL)	192.14 ± 40.86	177.84 ± 45.64	192.61 ± 40.61	< 0.001
Triglyceride (mg/dL)	120.49 ± 68.46	138.43 ± 68.55	119.90 ± 68.38	< 0.001
Sex, *n* (%)				
Male	4131 (47.64)	143 (3.46)	3988 (96.54)	< 0.001
Female	4540 (52.36)	134 (2.95)	4406 (97.05)	
Race, *n* (%)				
Mexican American	1234 (14.23)	16 (1.30)	1218 (98.70)	< 0.001
Other Hispanic	874 (10.08)	25 (2.86)	849 (97.14)	
Non‐Hispanic White	4105 (47.34)	162 (3.95)	3943 (96.05)	
Non‐Hispanic Black	1656 (19.10)	65 (3.93)	1591 (96.07)	
Other race—including multiracial	802 (9.25)	9 (1.12)	793 (98.88)	
Education, *n* (%)				
Less than 9th grade	756 (8.71)	38 (5.03)	718 (94.97)	< 0.001
9–11th grade (includes 12th grade with no diploma)	1199 (13.82)	59 (4.92)	1140 (95.08)	
High School Grad/GED or Equivalent	1946 (22.44)	70 (3.59)	1876 (96.41)	
Some College or AA degree	2538 (29.27)	75 (2.95)	2463 (97.05)	
College Graduate or above	2232 (25.74)	35 (1.57)	2197 (98.43)	
Matrimony, *n* (%)				
Married	4606 (53.12)	134 (2.91)	4472 (97.09)	< 0.001
Widowed	687 (7.92)	64 (9.32)	623 (90.68)	
Divorced	937 (10.81)	45 (4.80)	892 (95.20)	
Separated	256 (2.95)	8 (3.13)	248 (96.88)	
Never married	1526 (17.60)	16 (1.05)	1510 (98.95)	
Living with partner	659 (7.60)	10 (1.52)	649 (98.48)	
PIR, *n* (%)				
≤ 1	1790 (20.64)	77 (4.30)	1713 (95.70)	< 0.001
1–3	3608 (41.61)	145 (4.02)	3463 (95.98)	
> 3	3273 (37.75)	55 (1.68)	3218 (98.32)	
BMI, *n* (%)				
≤ 19.9	365 (4.21)	7 (1.92)	358 (98.08)	< 0.001
19.9–24.9	2159 (24.90)	52 (2.41)	2107 (97.59)	
25–29.9	2891 (33.34)	76 (2.63)	2815 (97.37)	
> 30	3256 (37.55)	142 (4.36)	3114 (95.64)	
Hypertension, *n* (%)				
Yes	3251 (33.64)	223 (6.86)	3028 (93.14)	< 0.001
No	5420 (66.36)	54 (1.00)	5366 (99.00)	
Diabetes, *n* (%)				
Yes	1104 (9.30)	103 (9.33)	1001 (90.67)	< 0.001
No	7364 (88.58)	165 (2.24)	7199 (97.76)	
Borderline	203 (2.12)	9 (4.43)	194 (95.57)	
Coronary heart disease, *n* (%)				
Yes	367 (4.23)	105 (28.61)	262 (71.39)	< 0.001
No	8304 (95.77)	172 (2.07)	8132 (97.93)	
Angina pectoris, *n* (%)				
Yes	216 (2.49)	60 (27.78)	156 (72.22)	< 0.001
No	8455 (97.51)	217 (2.57)	8238 (97.43)	
Heart disease, *n* (%)				
Yes	371 (4.28)	118 (31.81)	253 (68.19)	< 0.001
No	8300 (95.72)	159 (1.92)	8141 (98.08)	
Apoplexy, *n* (%)				
Yes	329 (3.79)	58 (17.63)	271 (82.37)	< 0.001
No	8342 (96.21)	219 (2.62)	8123 (97.38)	
Cancer, *n* (%)				
Yes	847 (9.77)	61 (7.20)	786 (92.80)	< 0.001
No	7824 (90.24)	216 (2.76)	7608 (97.24)	
Smoking, *n* (%)				
Yes	3862 (44.54)	157 (4.07)	3705 (95.93)	< 0.001
No	4809 (55.46)	120 (2.49)	4689 (97.51)	
Tipple, *n* (%)				
Yes	6281 (72.44)	183 (2.91)	6098 (97.09)	< 0.001
No	2390 (27.56)	94 (3.93)	2296 (96.07)	
Sleep, *n* (%)				
≤ 7	5269 (60.77)	157 (2.98)	5112 (97.02)	< 0.001
7–8	2423 (27.94)	75 (3.10)	2348 (96.90)	
> 8	979 (11.29)	45 (4.60)	934 (95.40)	

*Note:* For continuous data, ANOVA was used to calculate *p* values, while categorical variables were assessed using chi‐square tests. A greater DI‐GM score reflects a more balanced and beneficial gut microbiome composition.

Abbreviations: BMI, body mass index; DI‐GM, the dietary index for gut microbiota; PIR, poverty income ratio.

### 3.2 Organization of the DI‐GM and CHF

As stated by Table 2's findings from the logistic regression analysis, higher DI‐GM scores in the unadjusted model had a negative correlation with the likelihood of developing congestive heart failure (CHF). Specifically, when comparing the first quartile (Q1) to the fourth quartile (Q4) (OR: 0.001, 95% CI: 0.0001–0.009), a much reduced chance of developing CHF was observed. In the initial model, after controlling to address possible factors that cause confusion such as age, sex, and race, the reduced likelihood of CHF persisted with incrementally higher DI‐GM scores. Notably, a significant inverse correlation was identified for Q4 (OR: 0.001, 95% CI: 0.0001–0.010) when compared to Q1. The subsequent model, which included further adjustments for a comprehensive array of covariates, the examination of multivariate logistic regression confirmed that a lower risk of CHF was nevertheless substantially connected to greater DI‐GM scores (OR: 0.0002, 95% CI: 0.000–0.001). However, unweighted results (Table S2) indicate that in Model 1, higher DI‐GM scores were associated with CHF (OR: 0.631, 95% CI: 0.440, 0.906). There is a nonlinear link between DI‐GM and CHF, according to the linear regression correlation data (Table S1).

**TABLE 2:** Logistic regression association between DI‐GM and CHF.

	Crude model OR 95% CI	*p*	Model 1 OR 95% CI	*p*	Model 2 OR 95% CI	*p*
Exposures DI‐GM	0.956 (0.877, 1.040)	0.310	0.922 (0.845, 1.010)	0.071	1.010 (0.906, 1.130)	0.846
Q1	Ref		Ref		Ref	
Q2	1.140 (0.760, 1.720)	0.513	1.010 (0.730, 1.390)	0.962	0.965 (0.698, 1.330)	0.827
Q3	1.220 (0.798, 1.880)	0.348	0.850 (0.590–1.230)	0.379	0.960 (0.676, 1.370)	0.820
Q4	0.001 (0.0001, 0.009)	< 0.001	0.001 (0.0001, 0.010)	< 0.001	0.0002 (0.000, 0.001)	< 0.001
*p* for trend		< 0.001		< 0.001		< 0.001

*Note:* Crude model did not adjust for covariates; Model 1 adjusted for age, sex, race; Model 2 adjusted for age, sex, race, education, matrimony, PIR, BMI, hyperlipidemia, hypertension, diabetes, coronary heart disease, angina pectoris, heart disease, apoplexy, cancer, sleep, smoking, tipple.

### 3.3 Nonlinear Patterns of the DI‐GM and CHF

**FIGURE 2:**
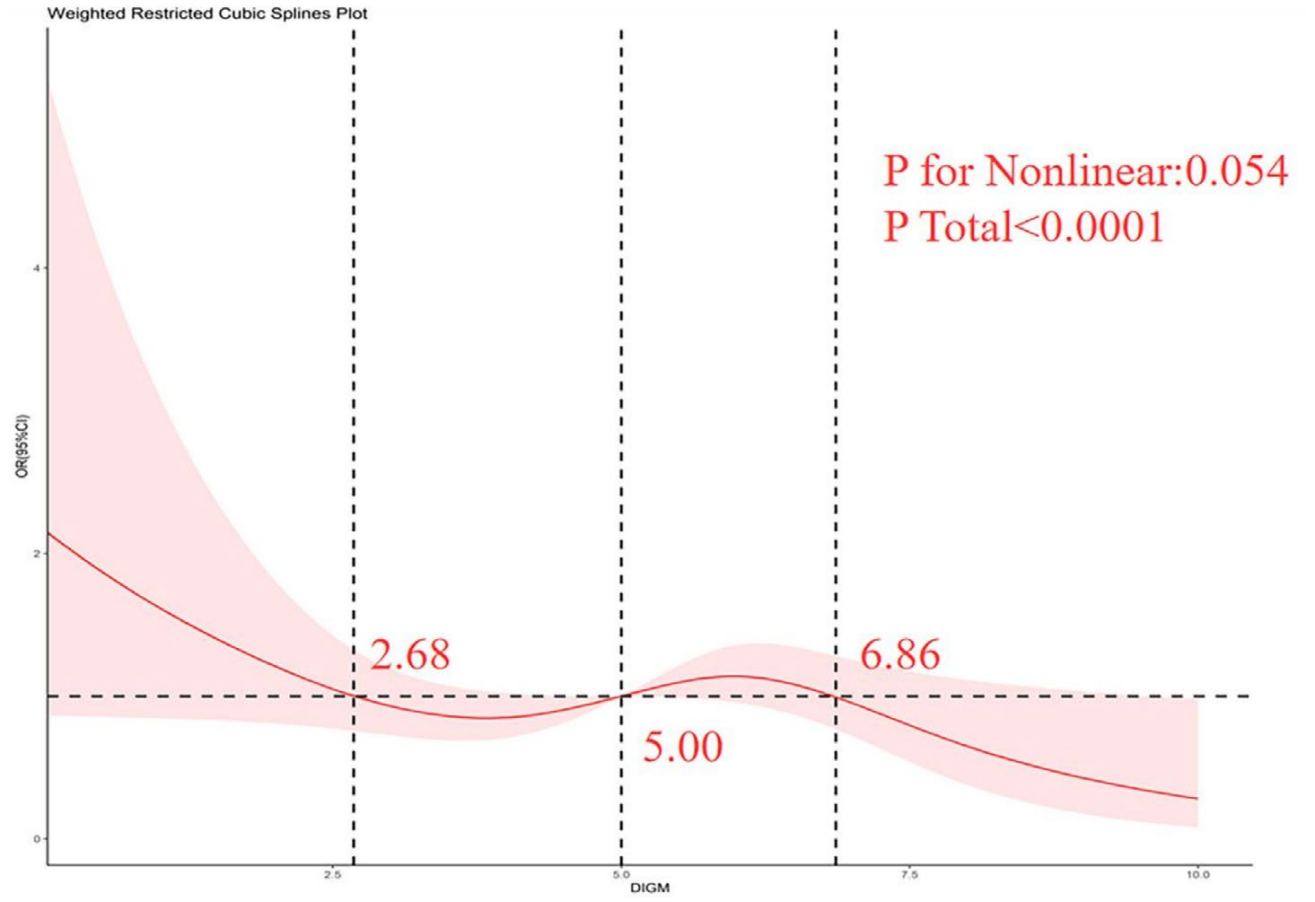
Connection between the DI‐GM and the CHF through the RCS.

We used an unrestricted cubic bar chart (RCS) in our analysis to understand whether there was an asymmetrical correlation between CHF and DI‐GM. As seen in Figure 2 and Figure S1, after adjustment for all covariates, there was a tendency for congestive heart failure to decrease with the rise in the DI‐GM score. We chose the point with an OR of 1 as the inflection point for congestive heart failure, and there were three inflection points, 2.68, 5.00, and 6.86. There was a nonlinear relationship between congestive heart failure and DI‐GM ratings, and we found that the danger of suffering from congestive heart failure began to increase in cases where the DI‐GM score was below 2.68 or was between 5.00–6.86 and that DI‐GM belonged to the category of risk factor; however, when the DI‐GM score was between 2.68–5.00 or greater than 6.86, the risk of developing congestive heart failure showed a decline, with DI‐GM acting as a protective element.

### 3.4 Subgroup Analyses

**FIGURE 3:**
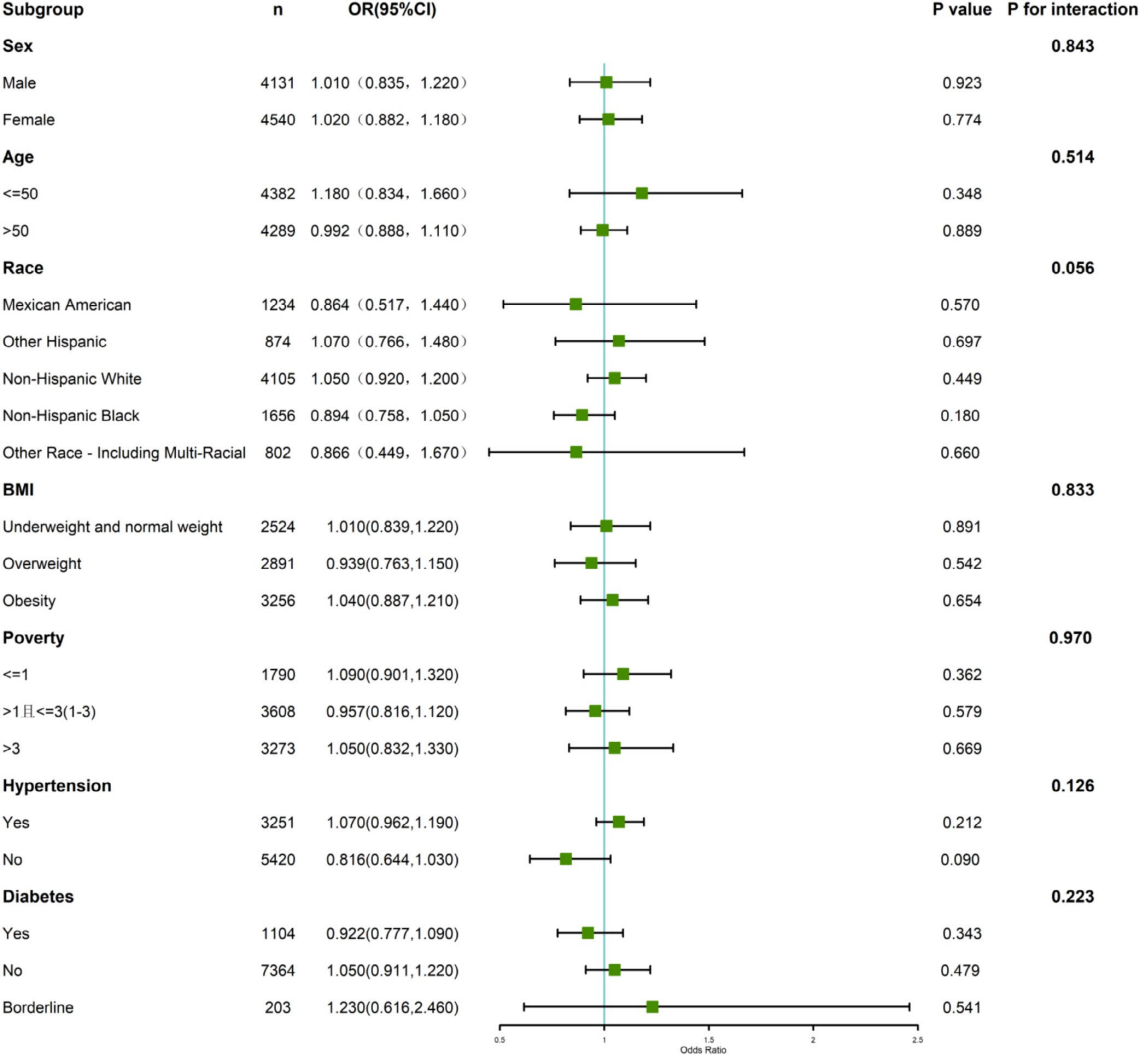
Stratified examination of the relationship between DI‐GM and CHF.

**FIGURE 4:**
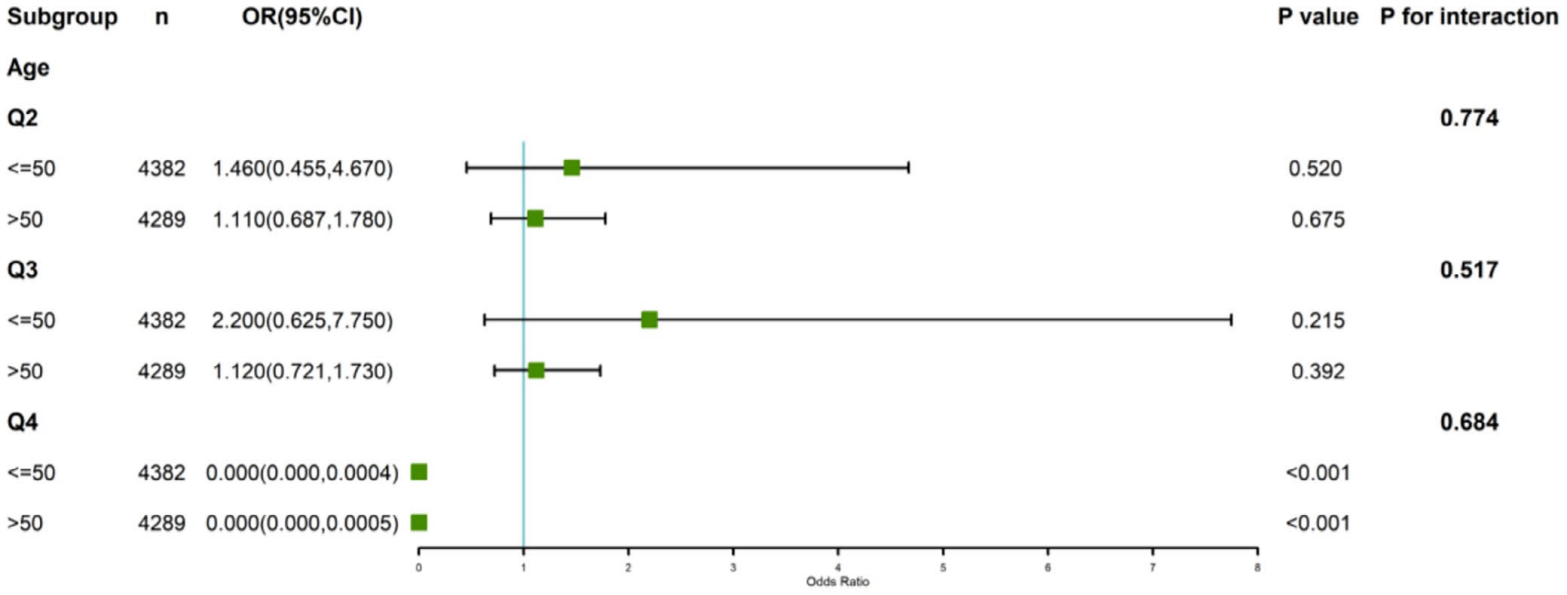
Stratified examination of the relationship between DI‐GM and CHF by age.

We stratified the overall population by gender, age, ethnicity, BMI, poverty status, hypertension, and diabetes, and conducted subgroup analyses. As seen in Figure 3 and Figure S2, we could not find any noteworthy correlation between continuous DI‐GM scores and the possibility of congestive heart failure throughout different subgroups. Furthermore, no important exchange between DI‐GM and congestive heart failure danger was noted in any of the seven subgroup analyses (*p* > 0.05). However, we further investigated the connection between age and categorized DI‐GM scores with the danger of congestive heart failure. As seen in Figure 4, we found that within age subgroups, higher DI‐GM ratings were connected to a significantly decreased chance of developing congestive heart failure compared to Q1 (*p* < 0.001 for age ≤ 50; *p* < 0.001 for age > 50). Furthermore, no noteworthy engagement between different DI‐GM groups and the danger of congestive heart failure was observed (*p* > 0.05).

### 3.5 Sensitivity Analysis

Our investigations, which integrated albumin concentrations in evaluating the danger of congestive heart failure, revealed a distinct trend across various analytical frameworks. By incorporating albumin into the assessment of congestive heart failure, our findings indicated that individuals exhibiting elevated DI‐GM scores demonstrated a markedly reduced likelihood of developing congestive heart failure when contrasted with the first quartile in the unadjusted model, as well as in Models 1 and 2 (Table 3). This observation aligns with the outcomes of our primary study. Moreover, in the unweighted results (Table S3), we observed that higher DI‐GM scores were associated with CHF in Model 1. According to the results of our rescheduling, participants with higher DI‐GM scores in Model 2 still had a significantly lower risk of congestive heart failure than in Q1, even after the addition of new covariates like physical activity, total energy intake, CKD, and related medications like diuretics, ACEi/ARB, and beta‐blockers (Table 4). Show that the outcomes of various treatments don't change.

**TABLE 3:** Association between DI‐GM and CHF (including albumin).

	Crude model OR 95% CI	*p*	Model 1 OR 95% CI	*p*	Model 2 OR 95% CI	*p*
Exposures DI‐GM	0.956 (0.877, 1.040)	0.310	0.922 (0.845, 1.010)	0.071	1.020 (0.917, 1.140)	0.679
Q1	Ref		Ref		Ref	
Q2	1.140 (0.760, 1.720)	0.513	1.010 (0.730, 1.390)	0.962	1140 (0.758, 1.720)	0.517
Q3	1.220 (0.798, 1.880)	0.348	0.850 (0.590–1.230)	0.379	1.280 (0.837, 1.960)	0.598
Q4	0.001 (0.0001, 0.009)	< 0.001	0.001 (0.0001, 0.010)	< 0.001	0.001 (0.000, 0.006)	< 0.001
*p* for trend		< 0.001		< 0.001		< 0.001

*Note:* Crude model did not adjust for covariates; Model 1 adjusted for age, sex, race; Model 2 adjusted for age, sex, race, education, matrimony, PIR, BMI, hyperlipidemia, hypertension, diabetes, coronary heart disease, angina pectoris, heart disease, apoplexy, cancer, sleep, smoking, tipple, albumin.

**TABLE 4:** Association between DI‐GM and CHF (including physical activity, total energy intake, CKD, related medications such as diuretics, ACEi/ARB, and β‐blockers).

	Crude model OR 95% CI	*p*	Model 1 OR 95% CI	*p*	Model 2 OR 95% CI	*p*
Exposures DI‐GM	0.940 (0.864, 1.022)	0.152	0.910 (0.843, 0.982)	0.020	0.000 (0.000, 0.000)	< 0.001
Q1	Ref		Ref		Ref	
Q2	0.577 (0.395, 0.844)	0.006	0.554 (0.379, 0.810)	0.004	0.000 (0.000, 0.000)	< 0.001
Q3	0.692 (0.418, 1.146)	0.158	0.712 (0.418, 1.211)	0.215	0.000 (0.000, 0.000)	< 0.001
Q4	0.670 (0.481, 1.017)	0.066	0.621 (0.425, 0.908)	0.017	0.000 (0.000, 0.000)	< 0.001
*p* for trend		< 0.001		< 0.001		< 0.001

*Note:* Crude model did not adjust for covariates; Model 1 adjusted for age, sex, race; Model 2 adjusted for age, sex, race, education, matrimony, PIR, BMI, hyperlipidemia, hypertension, diabetes, coronary heart disease, angina pectoris, heart disease, apoplexy, cancer, sleep, smoking, tipple, physical activity, total energy intake, CKD, related medications such as diuretics, ACEi/ARB, and β‐blockers.

## 4 Discussion

Using NHANES data, we investigated the connection between congestive heart failure and DI‐GM scores. Groups of participants were formed according to whether or not they had congestive heart failure. DI‐GM levels and the danger of developing CHF were found to be significantly inversely correlated by our logistic regression analysis. The model of the limited cubic spline showed a consistent dose–response pattern, where progressively higher DI‐GM quartiles corresponded to substantially reduced risk when measured against the baseline reference group (Q1). These outcomes show that elevated DI‐GM ratings are related to reduced susceptibility to heart failure, providing new insights into nutritional strategies and preventive measures for this cardiovascular disease.

As outlined by the American Heart Association and the American College of Cardiology (AHA/ACCF), CHF is characterized as “a multifaceted clinical syndrome that may arise devoid of any structural or functional cardiac disorder that hinders either the filling or ejection of the ventricles” (Kennelly et al. 2022). Congestive heart failure represents the last stage of cardiovascular disease and is a primary contributor to mortality rates. A novel perspective on diseases posits that a significant number of chronic conditions may originate in the gastrointestinal tract, after the release of various physiologically active compounds from the gut microbiota into the bloodstream and the body's circulatory system (Kase et al. 2024). For instance, trimethylamine, which is rapidly transformed to trimethylamine N‐oxide (TMAO) by hepcidin monooxygenase 3, is produced during the intestinal microbial metabolism of dietary nutrients. Various datasets indicate a significant correlation between gut microbiota formation of the metabolite TMAO and adverse cardiovascular risk (Organ et al. 2016). In turn, gut microbes create vast quantities of short‐chain fatty acids (SCFA), oxidized trimethylamine, indole sulfates, and bile acid content through glucose metabolism and amino acid metabolism, which can lead to the heterogeneity of inflammatory products in the heart system, resulting in symptoms of heart failure (Li et al. 2025). Reduced scores on the DI‐GM scale have been correlated with a heightened likelihood of developing congestive heart failure. This association may be partly attributed to the decrease in butyrate‐generating bacteria, which can impair the function pertaining to the intestinal mucosa. Such impairment can result in the passive translocation of microbial toxins, potentially contributing to the onset of inflammation‐driven diseases (Trøseid et al. 2020). A 2025 study by Fadhillah Farhan S. and colleagues (Fadhillah et al. 2025) revealed that prolonged intake of carbohydrate‐rich foods could disrupt gut microbiota balance, leading to elevated Prevotella levels and reduced Anaplasma populations. Additionally, the research demonstrated that high‐fat, low‐fiber diets compromise intestinal health by weakening gut barrier integrity and inducing unfavorable shifts in microbial composition. Therefore, studying the connection between dietary indices of gut microbiota and CHF, and maintaining the amount and variability of gut microbiota and its metabolites present in vivo (Li et al. 2025) may supply fresh concepts and contribute positively to the therapy of CHF.

By generating a variety of metabolites, the microbiota in the gut may either directly or indirectly have an impact the health of the metabolism of the host. Trimethylamine (TMA), which is created by gut bacteria from the diet nutrients, is oxidized by flavin inside the liver monooxygenase to trimethylamine N‐oxide (TMAO) once it enters the bloodstream (Zhang et al. 2021). The TMAO pathway could immediately lead to unfavorable ventricular remodeling and the emergence of heart failure phenotypes, and increased levels of TMAO are connected to a higher danger of CHF. Heart failure may result from this pathway's exacerbation of chamber dilatation, wall thinning, decreased shortening fraction, and increased fibrosis (Tang et al. 2017). Studies on fecal microbiota transplantation show that microbial community transfer may restore the pro‐atherosclerotic characteristics caused by TMAO (Shen et al. 2025). SCFAs generated by the gut microbiota may work by promoting the infiltration of CX3CR1+ macrophages in the peri‐infarct area, which is essential for healing cardiac damage after an infarction. Short‐chain fatty acids may thereby stop inflammation from starting and spreading, which might result in heart failure (Chen, Zhang, et al. 2023). Characteristics of the microbiome in the gut show up as increased permeability of the intestines, poor microcirculation, and intestinal wall hyperemia and edema among individuals with cardiac cachexia and chronic heart failure. This results in the movement of microorganisms and their byproducts, as well as a dysbiosis dominated by Firmicutes, Bacteroidetes, and Proteobacteria, which may have an effect on cardiovascular health (Nesci et al. 2023).

Subgroup and interaction analyses played a significant part in delving into the associative aspects of the factors. The study's findings revealed significant differences between DI‐GM and CHF in various age groups. A higher risk of developing CHF with DI‐GM scores was observed in people above 50 years of age. This observation could be the result of aging‐related alterations in the gut flora (Chen, Wang, et al. 2023). Kamo et al. (2017) found that the proportion of *Anaplasma* spp. Decreased the number of *Aspergillus* spp. increased among heart failure patients in their senior years. *E. faecalis* spp. were exhausted, while *Lactobacillus* spp. were enriched in older heart failure patients' gut microbiota. Gut microbial dysbiosis can cause the evolution of systemic inflammation, which is connected to age‐related declines within tissue and immune function (Lu et al. 2025). In 2020, Golomb et al. (2020) found that systemic changes that can be induced by aging and dysbiosis of gut ecology increased the inclination towards neuroinflammation, which offered a valuable understanding of the dysbiosis of gut ecology in neurological disorders connected with aging.

The dietary index of gut microbiota (DI‐GM) encapsulates the possible correlation between nutritional patterns and the variety of microbiome in the gut (Zheng et al. 2025). For patients with cardiovascular disease, dietary rationalization and optimization of dietary components are especially crucial to preserve good health (Jin et al. 2019) Fermented dairy items are unique and advantageous elements of DI‐GM, containing beneficial microorganisms, microbial metabolites, and bioactive (Liu and Huang 2025). In 2018, Zhang et al. (2020) found by Meta‐analysis that the intake of fermented the intake of dairy products has been connected to a decreased likelihood of heart conditions. β‐Accompanied soy protein is the main component of soy protein. Furukawa et al. (2024) found that beta‐associated protein from soy increased the gut microbiota producing SCFAs and intestinal SCFAs and improved the course of left ventricular renovating in aortic constriction‐mediated heart failure. Grains contain important nutrients such as vitamins, minerals, fiber, and oils. Hu et al. (2023) found by Meta‐analysis that the intake of entire grains, instead of refined cereals, may help prevent cardiovascular disease (CVD), coronary heart disease (CHD), and all‐cause mortality. Djoussé and Gaziano (2007) showed by a prospective follow‐up study that greater usage of whole grain cereals for breakfast was linked to a reduced danger of heart failure. Unhealthy dietary patterns are one of the risk factors for disorders of energy metabolism and further development of cardiovascular disease (Videja et al. 2020). In the Western diet, Fabiana A. Neves et al. (Neves et al. 2014) found that elevated consumption of fats, red meat, and processed food items impaired cardiac bioenergetic metabolism by reducing mitochondrial biosynthesis and mitochondrial uncoupling, leading to impaired myocardial oxidative capacity, and Liu et al. (2023) discovered that a diet heavy in fat produced high the concentrations of Beta‐interleukin‐1 (IL‐1β) and proinflammatory proteins in the heart and accumulation of pro‐inflammatory cardiac macrophages, leading to diastolic dysfunction. These studies all highlight the twofold function of diet and microbiome in the gut in the avoidance of CHF. In addition, dietary intake modulates plasma redox and protects against reactive oxygen species, and the negative connections between DI‐GM and congestive heart failure is dependable with the risk of heart failure associated with Dietary Antioxidant Composite Index (CDAI) (Ma et al. 2023). The DI‐GM presents a fresh method for comprehending the connection between diet and CHF, complementing indices such as the CDAI, and has the potential to confirm in further studies the causality before mitigating the risk of congestive heart failure.

Research indicates that numerous dietary elements rely on gut microbiota for proper metabolic processing, with significant variations observed among individuals in how these microbial communities generate metabolites linked to health outcomes (Meyer and Bennett 2016). In a 2024 study, Mingming Luo and colleagues (Luo et al. 2024) proposed that dietary copper intake may influence human health through interactions with gut bacteria. Based on these findings, the researchers recommended that Chinese nutritional guidelines might benefit from revising downward the maximum recommended daily copper allowance for women in their years of reproduction. This adjustment would account for the potential health implications of copper's effects on the microbiome. Marques et al. (2017) discovered that a significant intake of fiber led to alterations to the intestinal microbiota, resulting in an increased population of bacteria that produce acetate. This alteration subsequently served as a safeguard against the beginning of cardiovascular disease. Some researchers argue that reducing inflammation and improving diet are key to chronic disease prevention and management (Wang et al. 2024). For example, appropriate supplementation with probiotics has the potential to modify both the functionality and the makeup of the intestinal microbiome, subsequently stimulating the immune response and regulating inflammatory processes (Jin et al. 2019), whereas the majority of prebiotics are naturally occurring carbs discovered in sources including veggies, fruits, and grains. In older people, there is a decline in gut function and a decrease in beneficial gut flora with a higher proportion of pro‐inflammatory bacteria (Matacchione et al. 2024), therefore, the intake of foods that are rich in beneficial gut microbiota can enhance nutrient absorption and strengthen the immune system (Kase et al. 2024), but in younger people, their gut function is relatively healthy without much noticeable changes. Therefore, for the prevention of heart‐related conditions such as CHF, measures can be developed based on individualized and age‐appropriate measures to prevent and improve gut flora to achieve a lower risk of developing CHF.

A key strength of our study is leveraging NHANES data with a large, representative sample, enhancing result generalizability. In addition, we implemented rigorous participant selection criteria and used a variety of statistical analyses to enhance the reliability and stability of our results. However, this research is constrained by various limitations. First, due to the cross‐sectional design, determining causality was challenging. Despite what we saw a notable correlation between DI‐GM and congestive heart failure, it does not imply an exact causal relationship. Secondly, the DI‐GM scores that we gathered and computed were derived from 24‐h dietary recall interviews, which were carried out either at a mobile screening facility or through telephone interviews. It is crucial to acknowledge that these approaches are prone to remember prejudice. Third, despite being a novel measure, the DI‐GM score was calculated to include only foods associated with the gut microbiota and did not include other foods that have not yet been explored. Fourth, insufficient reporting, erroneous information, and poor persuasiveness will result from the NHANES database's determination of CHF based on whether the patient has ever been told they had it. Fifth, a reverse causal link might result from BMI acting as a mediator mediating the relationship between DI‐GM and CHF. We investigated other markers including waist circumference and C‐reactive protein, but the causal association was not evident. As a result, the mediating correlation analysis was eliminated. Finally, the sample in this study consisted primarily of non‐institutionalized civilians in the USA, which might restrict the findings' generalisability to other nations. Therefore, future longitudinal follow‐up studies could be conducted to look into the potential causal hyperlink between DI‐GM and the onset of congestive heart failure.

## 5 Conclusion

This research leveraged NHANES data spanning spanning 2007–2018 to investigate the partnership between DI‐GM and congestive heart failure (CHF). The examination showed a strong inverse correlation—higher DI‐GM values were consistently connected to a decreased danger of CHF. This association kept firm even when accounting for confounding variables, demonstrating remarkable resilience in the statistical model. Interestingly, the connection followed a nonlinear pattern rather than a straightforward linear trend. To build on these insights, future longitudinal studies could track DI‐GM fluctuations over time, offering a clearer picture of how dynamic changes might impact CHF development.

## Author Contributions

**Yu Fu:** data curation (lead), methodology (lead), software (lead), writing – original draft (equal). **Hanfei Wang:** validation (equal). **Yulu Jiang:** validation (equal), writing – original draft (equal). **Jiana Yang:** validation (equal), writing – original draft (equal). **Jianquan Yu:** formal analysis (lead). **Anru Cao:** investigation (lead). **Yue Liu:** conceptualization (equal), visualization (lead). **Jie Wu:** conceptualization (equal), resources (lead), writing – review and editing (lead).

## Funding

This research was funded by NAME OF FUNDER, Liaoning Provincial Department of Education in 2023 (Grant No. JYTMS20231389); Liaoning Provincial Science and Technology Department joint fund (Grant No. 2023‐MSLH‐297, 2024JH2/102600235); 2024 Shenyang Medical College Graduate Student Scientific and Technological Innovation Fund Project (Y20240533); Shenyang Youth and Middle‐aged Science and Technology Innovation Talent Special Program (RC240110).

## Disclosure

Institutional Review Board Statement: “Not applicable” refers to research that does not use people or animals.

## Consent

The authors have nothing to report.

## Conflicts of Interest

The authors declare no conflicts of interest.

## Supporting information


**Table S1:** Linear regression association between DI‐GM and CHF.
**Table S2:** Unweighted logistic regression association between DI‐GM and CHF.
**Table S3:** Unweighted analysis of the association between DI‐GM and CHF (including albumin).
**Figure S1:** Unweighted analysis of the nonlinear relationship between DI‐GM and CHF.
**Figure S2:** Unweighted stratified examination of the relationship between DI‐GM and CHF.

## Data Availability

The data supporting the study's conclusions may be obtained upon request from the corresponding author (wujie073@163.com). The data are not publicly available due to privacy and ethical restrictions.
