# Supplementary material for: Relationship Between Congestive Heart Failure and the Dietary Index of Gut Microbiota: Information From the National Health and Nutrition Examination Survey 2007–2018
Source: Food Sci Nutr. 2025 Dec 31;14(1):e71386. doi: 10.1002/fsn3.71386 (PMC12754692; doi:10.1002/fsn3.71386)
Supplement: Supplementary file 1 — Table S1: Linear regression association between DI‐GM and CHF. Table S2: Unweighted logistic regression association between DI‐GM and CHF. Table S3: Unweighted analysis of the association between DI‐GM and CHF (including albumin). Figure S1: Unweighted analysis of the nonlinear relationship between DI‐GM and CHF. Figure S2: Unweighted stratified examination of the relationship between DI‐GM and CHF. [file FSN3-14-e71386-s001.docx]

| **Exposures DI-GM** | **β** | ***P* value** | **β 95% CI** |
| --- | --- | --- | --- |
| Crude model | -0.001 | 0.272 | -0.001(-0.003, 0.001) |
| Model 1 | -0.002 | 0.014 | -0.002(-0.004,-0.000) |
| Model 2 | 0.0005 | 0.611 | 0.0005(-0.001,0.002) |

**Table S1.** Linear regression association between DI-GM and CHF.

Crude model did not adjust for covariates;

Model 1 adjusted for age, sex, race;

Model 2 adjusted for age, sex, race, education, matrimony, PIR, BMI, hyperlipidemia, hypertension, diabetes, coronary heart disease, angina pectoris, heart disease, apoplexy, cancer, sleep, smoking, tipple

**Table S2.** Unweighted logistic regression association between DI-GM and CHF.

|  | **Crude model OR 95% CI** | **P value** | **Model 1**  **OR 95% CI** | **P value** | **Model 2**  **OR 95% CI** | **P value** |
| --- | --- | --- | --- | --- | --- | --- |
| Exposures DI-GM | 0.914(0.851,0.981) | 0.001 | 0.889(0.827,0.955) | 0.001 | 0.958(0.883,1.040) | 0.302 |
| Q1 | Ref |  | Ref |  | Ref |  |
| Q2 | 0.956(0.703,1.300) | 0.773 | 0.963(0.704,1.320) | 0.816 | 1.050(0.743,1.490) | 0.774 |
| Q3 | 0.966(0.692,1.350) | 0.837 | 0.924(0.658,1.300) | 0.649 | 1.200(0.820,1.750) | 0.351 |
| Q4 | 0.741(0.519,1.060) | 0.097 | 0.631(0.440,0.906) | 0.013 | 0.869(0.578,1.310) | 0.501 |
| P for trend |  | <0.001 |  | <0.001 |  | <0.001 |

Crude model did not adjust for covariates;

Model 1 adjusted for age, sex, race;

Model 2 adjusted for age, sex, race, education, matrimony, PIR, BMI, hyperlipidemia, hypertension, diabetes, coronary heart disease, angina pectoris, heart disease, apoplexy, cancer, sleep, smoking, tipple

**Figure S1.** Unweighted analysis of the nonlinear relationship between DI-GM and CHF.


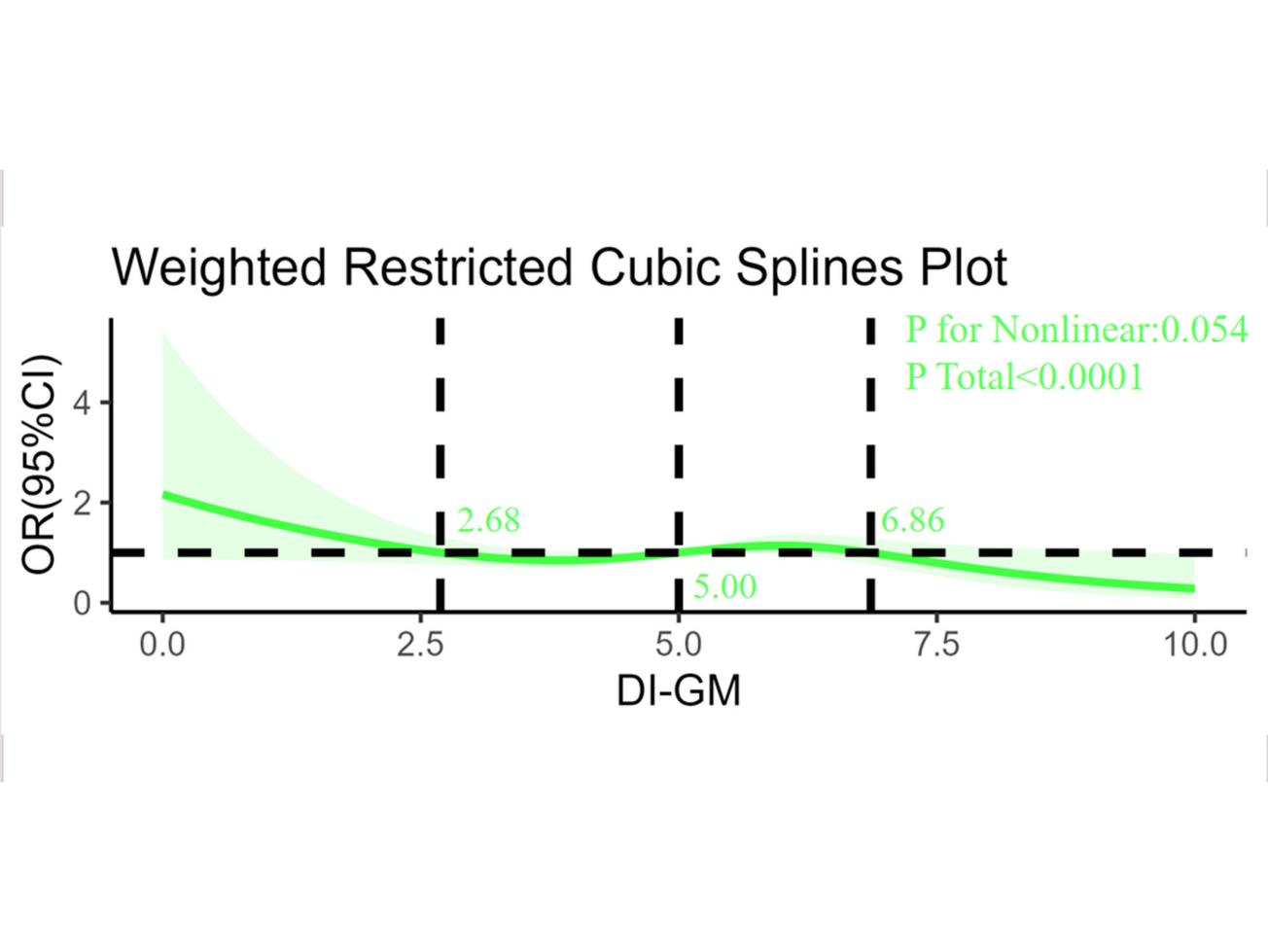


**Figure S2.** Unweighted stratified examination of the relationship between DI-GM and CHF

**Table S3.** Unweighted analysis of the association between DI-GM and CHF (including albumin)

|  | **Crude model OR 95% CI** | **P value** | **Model 1**  **OR 95% CI** | **P value** | **Model 2**  **OR 95% CI** | **P value** |
| --- | --- | --- | --- | --- | --- | --- |
| Exposures DI-GM | 0.914(0.851,0.981) | 0.013 | 0.889(0.827,0.955) | 0.001 | 10.969(0.893,1.050) | 0.450 |
| Q1 | Ref |  | Ref |  | Ref |  |
| Q2 | 0.956(0.703,1.300) | 0.773 | 0.963(0.704,1.320) | 0.816 | 1.060(0.748,1.500) | 0.744 |
| Q3 | 0.966(0.692,1.350) | 0.837 | 0.924(0.658,1.300) | 0.649 | 1.250(0.857,1.830) | 0.246 |
| Q4 | 0.741(0.519,1.060) | 0.097 | 0.631(0.440,0.906) | 0.013 | 0.908(0.603,1.370) | 0.646 |
| P for trend |  | <0.001 |  | <0.001 |  | <0.001 |

Crude model did not adjust for covariates;

Model 1 adjusted for age, sex, race;

Model 2 adjusted for age, sex, race, education, matrimony, PIR, BMI, hyperlipidemia, hypertension, diabetes, coronary heart disease, angina pectoris, heart disease, apoplexy, cancer, sleep, smoking, tipple, albumin
